# High Levels of Glycated Hemoglobin (HbA1c) Are Associated with Physical Inactivity, and Part of This Association Is Mediated by Being Overweight

**DOI:** 10.3390/nu15051191

**Published:** 2023-02-27

**Authors:** Samara Silva de Moura, Luiz Antônio Alves de Menezes-Júnior, Ana Maria Sampaio Rocha, Aline Priscila Batista, Mariana Carvalho de Menezes, Júlia Cristina Cardoso Carraro, George Luiz Lins Machado-Coelho, Adriana Lúcia Meireles

**Affiliations:** 1 Postgraduate Program in Health and Nutrition, Nutrition School, Federal University of Ouro Preto, Ouro Preto 35400000, MG, Brazil; 2 Epidemiology Laboratory, Medical School, Federal University of Ouro Preto, Ouro Preto 35400000, MG, Brazil; 3 Postgraduate Program in Biological Sciences, Federal University of Ouro Preto, Ouro Preto 35400000, MG, Brazil

**Keywords:** COVID-19, glycated hemoglobin, physical inactivity, chronic diseases, public health

## Abstract

The COVID-19 pandemic has generated substantial changes in the lives of the population, such as increased physical inactivity, which can lead to being overweight and, consequently, repercussions on glucose homeostasis. A cross-sectional study based on the adult population of Brazil was conducted by stratified, multistage probability cluster sampling (October and December 2020). Participants were classified as physically active or inactive during leisure time according to the recommendations of the World Health Organization. HbA1c levels were categorized as normal (≤6.4%) or with glycemic changes (≥6.5%). The mediating variable was being overweight (overweight and obese). Descriptive, univariate, and multivariate logistic regression analyses examined the association between physical inactivity and glycemic changes. Mediation was analyzed using the Karlson–Holm–Breen method to verify the influence of being overweight on the association. We interviewed 1685 individuals, mostly women (52.4%), 35–59 years old (45.8%), race/ethnicity brown (48.1%), and overweight (56.5%). The mean HbA1c was 5.68% (95% CI: 5.58–5.77). Mediation analysis verified that physically inactive participants during leisure time were 2.62 times more likely to have high levels of HbA1c (OR: 2.62, 95% CI: 1.29–5.33), and 26.87% of this effect was mediated by over-weight (OR: 1.30: 95% CI: 1.06–1.57). Physical inactivity at leisure increases the chances of high levels of HbA1c, and part of this association can be explained by being overweight.

## 1. Introduction

The COVID-19 pandemic promoted profound changes in the daily lives of citizens around the world; the social restriction, although necessary, negatively altered some health behaviors, especially those related to lifestyle habits, such as food consumption and physical activity practice [1]. Studies conducted during the pandemic showed that physical inactivity (PI) increases considerably during home confinement [2,3,4,5]. In addition, physical inactivity and the adoption of unhealthy eating habits may have influenced weight gain during the pandemic [6], which may have affected glycemic levels [7]. Although studies have shown the negative effects of the pandemic on the general health of the population [7,8], it is important to understand how physical inactivity during social restrictions can be associated with glycemic changes, thus posing a threat to the increased incidence of type 2 diabetes mellitus (T2DM) worldwide.

T2DM has emerged as a serious public health problem due to a modern lifestyle characterized by increased sedentary behavior and the consumption of ultra-processed foods [9]. The International Diabetes Federation has reported that approximately 422 million people worldwide live with diabetes mellitus [9,10]. One of the measures used to determine glycemic control is the HbA1c test, considered the gold standard for assessing glycemic control and an indicator of the average plasma glucose level during the previous 120 days [11].

Abnormalities in serum HbA1c concentrations are implicated in disease progression and physical disability, resulting in microvascular and macrovascular risks, neuropathy, retinopathy, renal dysfunction, and cardiovascular disease [9,12].

Some behaviors increase the risk of HbA1c changes, including smoking, poor eating habits, being overweight, and physical inactivity [13]. However, studies suggest that physical activity is an important non-pharmacological intervention for the treatment of diabetes mellitus, acting significantly on insulin resistance, glycemic control, and reducing HbA1c [14,15]. Therefore, the recommendation for adults to involve in regular physical activity is at least 150 min of moderate physical activity or 75 min of vigorous physical activity weekly [13].

Faced with the scarcity of biochemical data during the period of social restriction due to the COVID-19 pandemic and the prospect of intense modification in lifestyle and health behavior, especially physical activity, which may have influenced weight gain and, consequently, glycemic change, it is important to understand the mechanisms associated with this clinical condition to try to minimize negative health impacts, in addition to a higher prevalence of diabetes in the medium/long term. Therefore, this study aimed to evaluate serum HbA1c levels and their association with overweight-related physical inactivity. We hypothesized that high levels of HbA1c may be associated with physical inactivity at leisure during the social restriction and that being overweight may be a mediating variable in this association.

## 2. Materials and Methods

### 2.1. Study Design and Sampling

This was a cross-sectional study using data from the COVID-Inconfidentes project, a population-based household epidemiological survey conducted between October and December 2020 in two municipalities (Ouro Preto and Mariana) in Minas Gerais, Brazil. Participants eligible for the study were permanent residents of households in the urban area of the municipalities, aged 18 years or older, and who consented to participate in the study. Individuals with impaired cognitive function, difficulty answering the questionnaire, or inability to provide blood samples due to difficulties in venous access were excluded.

For data collection, a sample calculation was performed based on the 2010 population census for the urban area of each city, adopting a confidence level of 95% and an effect size equal to 1.5. Furthermore, 20% was added to the sample size of each city for possible refusals, absence of the resident selected, or people not at home during the visit. The sample size was calculated using OpenEpi (https://www.openepi.com/Menu/OE_Menu.htm, accessed on 7 February 2023), the minimum number of volunteers after the sample calculation for both municipalities (Ouro Preto and Mariana/MG) with 732 interviews, respectively. A three-stage sampling was used: census sector (selected with probability proportional to the number of households), household (selected from systematic sampling), and residents (≥18 years old, randomly selected by applying Sorteador de Nomes). The sample weight of each selected unit (census sector, household, and individual) was calculated to correlate with the 2019 population projections (DATASUS) [16]. In this calculation, adjustments were made to compensate for interview losses due to non-response. For more details on sample calculation and field logistics, see Meireles et al. 2021 [17].

### 2.2. Data Collection

In each municipality, data were collected on three weekends (Friday, Saturday, and Sunday), with intervals of 21 days between each. In the week preceding the data collection weekend, actions were carried out to disseminate the survey in selected census sectors, draw households, draw lots, and approach households to increase awareness and adherence to the survey. On the days of data collection, a resident was selected, followed by blood collection and face-to-face interviews. For venous blood collection, a 2.7 mL S-Monovette (Sarstedt) tube containing sodium fluoride/EDTA was used to analyze serum HbA1c levels.

Face-to-face interviews lasted 30 to 45 min, using the questionnaire in the DataGoal application via tablets. The interviewers maintained a minimum distance of 1.5 m from the interviewee as a protective measure against COVID-19, and physical contact was restricted to the point of collection of biological material.

The questionnaire included sociodemographic and economic variables, lifestyle habits, and general health status.

### 2.3. Outcome Variable: Glycated Hemoglobin (HbA1c)

HbA1c was measured in the Clinical Analysis Pilot Laboratory (LAPAC) of the School of Pharmacy/Federal University of Ouro Preto using the immunoturbidimetry method in the COBAS INTEGRA 400 plus automatic analyzer (Roche, Germany), following a protocol standardized by the manufacturer. Before each analysis, the device was calibrated with quality controls (HbA1c Control N and HbA1c Control P, Roche). A minimum volume of 400 µL of whole blood was used for the samples. The normal range adopted for the HbA1c level was ≤6.4%, and levels ≥ 6.5% were classified as having high levels of HbA1c [18].

### 2.4. Explanatory Variable: Physical Inactivity in Leisure Time

The self-reported physical activity during leisure time was evaluated by weekly frequency, duration, and type of physical exercise. Moderate physical activity was defined as walking, treadmill walking, weight training, water aerobics, Pilates, volleyball, and dancing, and vigorous physical activity included running, cycling, swimming, treadmill running, aerobics in general, wrestling, soccer/futsal, basketball, and tennis [19,20,21,22]. Then, the weekly frequency (0 to 7 days) was multiplied by the daily time (in minutes) to obtain the weekly amount of physical activity during leisure time in minutes. This amount was categorically evaluated using the cut-off points established by the World Health Organization (WHO) and the Physical Activity Guide for the Brazilian Population [23,24] into physically active (≥150 min/week of moderate physical activity, ≥75 min/week of vigorous physical activity, or a combination of both) or physically inactive (<150 min/week of moderate physical activity or <75 min/week of vigorous physical activity).

### 2.5. Mediating Variable: Overweight

Overweight was used as a mediation variable, measured by body mass index (BMI), calculated from self-reported weight and height according to the following formula: BMI = body weight (kg)/height (m^2^). kg/m^2^ if <60 years to WHO cut-off points into “not overweight” (BMI < 25.0 kg/m^2^ if <60 years or BMI < 28.0 kg/m^2^ if ≥60 years) and “overweight” (BMI ≥ 25.0 kg/m^2^ if <60 years or BMI ≥ 28.0 kg/m^2^ if ≥60 years) [25,26].

### 2.6. Adjustment of Variables

Sociodemographic covariates, tobacco use, and morbidities were included. The sociodemographic covariates determined were sex (women and men), age group (18–34 years; 35–59 years; ≥60 years), race/ethnicity categories were self-reported (white, black, brown, and others: yellow and indigenous), marital status (single or married), current family income (≤2 wages; >2 to ≤4 wages; >4 wages), and education (non-literate; <9 years; ≥9 years).

Tobacco consumption was determined using the question, “Do you smoke, or have you ever smoked cigarettes or any other tobacco product?” The answer options were yes and no.

Reported morbidity was determined by self-reporting the following diseases: high blood pressure, asthma, lung disease, chronic kidney disease, depression, anxiety disorder, obstructive sleep apnea, cancer, heart disease, or thyroid disease. Individuals who reported having at least one of the diseases were classified as having morbidity, and those with no disease had no morbidity.

### 2.7. Ethical Considerations

This study was approved by the Ethics Committee on Human Research of the Federal University of Minas Gerais (protocol number:32815620.0.1001.5149). All procedures followed the Brazilian guidelines and standards for human research. Participants were informed about the research objectives, the steps to be taken, and the risks and benefits of their participation. Those who agreed to participate signed an informed consent form.

### 2.8. Statistical Analysis

The study population was characterized using descriptive calculations, such as relative frequencies, mean values, and 95% confidence intervals (CI). Pearson chi-square test was used to verify the relationship between high levels of HbA1c and the sociodemographic characteristics of the study population. Univariate and multivariate logistic regression analyses assessed the association between physical inactivity and glycemic changes.

**Figure 1:**
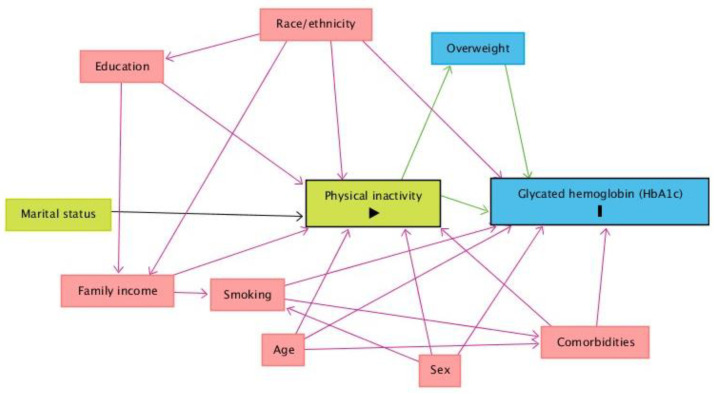
Directed acyclic graph (DAG) on glycated hemoglobin (HbA1c, outcome variable) and physical inactivity (exposure variable). Legend: The variable in green and with the symbol “>’’ inside the rectangle is the exposure variable. The variable in blue and with the letter “I” inside the rectangle is the outcome variable; Key: The variable with symbol “>’’ inside the rectangle is the exposure variable. In blue and with the letter I is the outcome variable; variables in blue are the antecedents of the outcome variable, and those in red are the antecedents of the outcome and exposure variables. Black arrows are non-causal and unbiased paths; green arrows are causal paths between the explanatory variable and outcome variable or antecedents; red arrows are biased paths.

A theoretical causality model based on a directed acyclic graph (DAG) was developed based on exposure (physical inactivity), outcome (glycemic changes, HbA1c ≥ 6.5%), and covariate variables using online Dagitty software, software version 3.2 [27] (Figure 1). DAG was used to avoid unnecessary adjustments, spurious associations, and estimation errors. The backdoor criterion selected a minimum set of confounders to fit the analyses [28]. The model was fitted using the following minimal and sufficient variables: sex, age, family income, race, and referred morbidities.

To verify whether overweight could be a mediating variable between the association of PI and glycemic changes, we used mediation analysis using the Karlson–Holm–Breen method, package “khb” in Stata [29]. This method estimates total, direct, and indirect associations between the explanatory variable (leisure time PI) and the outcome variable (glycemic changes: serum HbA1c levels ≥ 6.5%). Using logistic regression models, the method decomposed the total effect of a variable into a direct effect (direct association of leisure time physical inactivity and glycemic changes) and an indirect effect (the mediating effect of overweight on glycemic changes).

Results were analyzed using the Stata statistical program, version 15.1, operating the “svy” command, which considers a complex sample design. Statistical significance was established at *p* < 0.05.

## 3. Results

We evaluated 1685 individuals, most of whom were women (52.4%; 95% CI: 40.5–54.8), aged 35–59 years old (45.8%; 95% CI: 41.2–50.5), married (52.9%; 95% CI: 46.8–58.9), self-reported as brown (48.1%; 95% CI: 41.7–54.5), less than nine years of schooling (69.1%; 95% CI: 64.3–73.6), income less than or equal to two minimum wages (41.2%; 95% CI: 35.6–47.1), and overweight (56.5%; 95% CI: 49.8–63.0). In addition, most of the respondents reported having one or more morbidities (55.5%; 95% CI: 48.3–62.4), as shown in Table 1.

**Figure 2:**
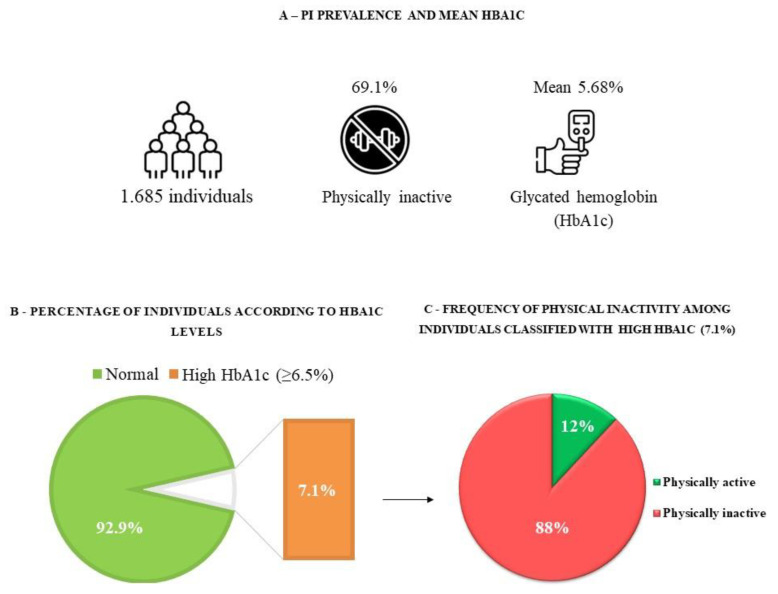
Serum HbA1c levels, prevalence of physical inactivity, and glycemic changes. COVID-Inconfidentes, October–December 2020.

The mean HbA1c was 5.68% (95% CI: 5.58–5.77). We observed that 92.9% (95% CI: 90.6–94.7) of the subjects had normal levels of HbA1c, and 7.1% (95% CI: 5.3–9.4) had glycemic changes. In addition, 69.2% (95% CI: 64.2–73.7) of the participants were physically inactive at leisure (Figure 2).

Table 1 presents the characteristics of the participants according to their serum HbA1c levels. Variables related to the presence of high levels of HbA1c according to the Pearson chi-square test (*p* < 0.05) were age ≥ 60 years, female, education ≤ 9 years, overweight, black race/ethnicity, and presence of morbidities (Table 1).

**Figure 3:**
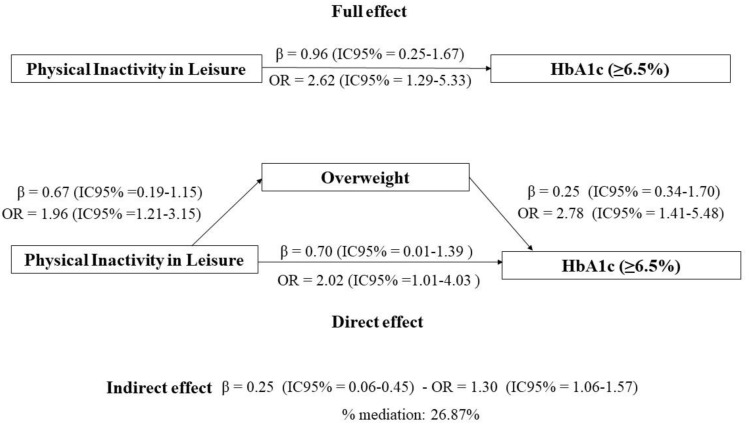
Mediation analysis of overweight between leisure time physical inactivity and high levels of HbA1c (≥6.5%) during the COVID-19 pandemic. Collection conducted in two Brazilian municipalities in the period October–December 2020. COVID-Inconfidentes, 2020. Legend: Explanatory variable; leisure time, physical inactivity. Outcome variable; glycated hemoglobin (HbA1c ≥ 6.5%). Mediating variable; overweight (BMI ≥ 25.0 kg/m^2^ if <60 years or BMI ≥ 27.0 kg/m^2^ if ≥60 years). Mediation model from Karlson–Holm–Breen method, adjusted according to directed acyclic graph according to minimum and sufficient set: age, sex, race/ethnicity, family income, and referred morbidity.

When examining the results of the logistic regression, it was observed in the multivariate analysis (adjusted for sex, age, race/ethnicity, family income, and reported morbidity) that individuals who were physically inactive during leisure time were 2.62 times more likely to have high levels of HbA1c (OR: 2.62, 95% CI: 1.31–5.24), as presented in Table 2. In the mediation analysis by overweight, it was possible to verify that physically inactive individuals during leisure time had 2.62 times greater chance of having high levels of HbA1c (OR: 2.62,95% CI: 1.29–5.33) and 26.87% of this effect was mediated by overweight (OR: 1.30, 95% CI: 1.06–1.57). The mediation calculation is obtained by total Beta (β) divided by indirect Beta (β), multiplied by 100. Therefore, β total/β indirect* 100 = mediated effect (Figure 3).

## 4. Discussion

This study investigated the association between physical activity and high levels of HbA1c, as well as the mediation of overweight in this association. Our findings confirm the initial hypothesis that adults who accumulate 150 min of moderate or 75 min of vigorous physical activity per week are less likely to have glycemic changes than individuals who do not reach the weekly minutes of physical activity recommended by the guidelines [23,24]. To our knowledge, this is the first major epidemiological survey to investigate HbA1c and physical inactivity during leisure time during social restrictions caused by the COVID-19 pandemic.

These results are important regarding public health and clinical practice, as approximately 70% of the population in this study did not meet the minimum guidelines for physical activity. Although our study did not allow us to assess the change in physical activity practice during the COVID-19 pandemic, it is believed that the pandemic may have contributed substantially to the increased prevalence of physical inactivity [3] due to the closure of recreational facilities, urban parks, and gyms to mitigate the spread of the virus. However, chronic damage caused by reduced levels of physical activity in the face of prolonged home stays has become a significant challenge [3,4]. The health consequences are inevitable, with deterioration of the physical and mental conditions of the population observed due to the confinement period [30].

Thus, some studies show a worsening prevalence of physical inactivity and overweight due to social restrictions and reduced urban mobility worldwide [4,6,31,32,33]. This panorama exacerbates the risks for the incidence and prevalence of chronic non-communicable diseases [34] and the increased occurrence of certain disorders, such as changes in HbA1c levels. These changes in glycemic homeostasis may reflect the progression and/or worsening of the clinical picture to type 2 diabetes mellitus [35]. Another concern is the susceptibility to infection and the risk of COVID-19 in individuals with high levels of HbA1c [36]. This highlights the importance of maintaining good control of serum Hb1Ac levels for overall health and to prevent worsening and complications associated with SARS-CoV-2 infection (Severe Acute Respiratory Syndrome Coronavirus 2).

Regular physical activity has long been considered a non-pharmacological way to improve general health, manage glycemic control, and treat T2DM, and its low cost has increased its appeal [37,38]. According to the American Diabetes Association [39], physical activity is essential for preventing the harmful effects of T2DM [40]. Musculoskeletal contraction stimulates the displacement of glucose transporter protein (GLUT-4) to the plasma membrane; this signaling promotes an acute increase in glucose transport and metabolization [15,41]. Thus, physical activity acts in several mechanisms that minimize the deleterious effects of high glucose levels, indirectly in fatty acid metabolism, helping the energy balance and consequently attenuating adiposity; being overweight is one of the main risk factors for the development of T2DM and, directly promotes improvement in glycemic control and insulin sensitivity [42].

A study of Brazilian women before (January and February 2020) and after 16 weeks of the COVID-19 pandemic (June and July 2020) showed that confinement promoted important changes in health-related parameters. HbA1c levels increased significantly by 9.7%, which was explained by reduced physical activity [35]. Consequently, a cross-sectional study conducted in India revealed the negative impact of social restrictions on glycemic control. The mean value of serum blood glucose concentrations worsened during the period of social restriction compared to the pre-pandemic time [7]. Furthermore, a systematic review concluded that the COVID-19 pandemic caused worsening glycemic control and complications related to T2DM; this observed worsening may be provinient to the closure of outdoor public places (such as parks and squares) that encourage the practice of physical activity [36].

Our results show an association between physical inactivity and high levels of HbA1c, and a part of this association can be explained by being overweight. These findings demonstrate clinical relevance, with physical activity effectively mitigating the risk of developing T2DM, both for body weight maintenance and through metabolic mechanisms that help glycemic control. A similar effect to ours was found in a systematic review and dose-response meta-analysis, which compared risk estimates of T2DM in relation to leisure time physical activity, with and without adjustment for the BMI covariate. The results with adjustment weakened the association by about 20%-30% when compared to the results unadjusted for BMI [42], thus demonstrating the mediating effect of BMI on the association between physical inactivity and glycemic changes.

Thus, a consistent justification for this association may be positive energy balance, that is, increased habitual food intake compared to the period before the COVID-19 pandemic (mainly of ultra-processed foods) and reduced energy expenditure by reducing urban mobility and physical activity. This may lead to weight gain and increase the risk of T2DM in individuals who are physically inactive [43,44]. Considering these findings, it is possible to postulate the lasting impact of the increased physical inactivity caused by the pandemic on the health of the population.

The findings of this study are of great value to the scientific community, leaders, and health professionals to better understand the side effects on short- and long-term health conditions of the COVID-19 pandemic and to devise strategies to mitigate these impacts. The COVID-19 pandemic may have provided a favorable scenario for morbidity and a poorer health prognosis. Furthermore, there are no structured educational programs for the self-management of T2DM in Brazil [45]. PA acts in the prevention and mitigation of complications from T2DM, in addition to mitigating the high costs to the public system, which is a burden on global health [12]. Health professionals operating in the treatment of T2DM should encourage the practice of PA, promoting the population’s understanding of the acute and chronic physiological responses to PA in T2DM.

Although robust and with relevant results in the scientific literature, this study had some limitations. The level of physical activity was obtained by self-reporting; therefore, it is subject to memory and other bias, which may underestimate or overestimate the data. Furthermore, calorie intake and added sugars, important covariates in the present association, were not assessed. Methodologically, the cross-sectional design of this study did not allow the establishment of causalities. However, using the counterfactual approach from directed acyclic graphs (DAG), it is possible to infer and verify the causal effect of associations from the observed variables and the assumptions in the diagram. It is emphasized that the hypotheses were carefully defined according to the current scientific literature and articulated in counterfactual terms to build theories that can underpin the driving assumptions of the analyses. In this regard, incorporating directed graphical models is of great importance and brings robustness to the study [46]. However, in observational studies, the assumptions necessary to estimate the causal effect are not empirically testable. Therefore, observational analyzes will always be subject to errors in the direction of the analysis.

In addition, our study has other strengths, such as a robust sample methodology with probability selection and sample weight, providing statistical power to the study. Furthermore, the interviews were conducted face-to-face, allowing greater accuracy in the information obtained. In addition, assessing glycemic homeostasis through Hb1Ac is noteworthy, as it represents glycemic levels during the last four months and not only at the current time.

## 5. Conclusions

Leisure time physical activity is associated with greater high levels of HbA1c and part of this association is explained by being overweight. Therefore, our data suggest that physical activity may attenuate the risk of developing T2DM, partly by controlling overweight but also independently of adiposity. In addition, glycemic changes are metabolic changes that require regular attention, given their implications for the worsening of population health and high costs to the public health system. Therefore, we believe that maintaining regular physical activity is essential for the management of glycemic control in periods of restricted social mobility.

During the pandemic, there was a great deal of uncertainty and fear around physical activity, especially in open areas, since little was known about the forms of contamination. This favored an increase in physical inactivity during data collection. However, with current knowledge, encouraging physical activity in open environments should be encouraged in times of restricted social mobility. Taking the necessary precautions to reduce contagion, or at least the encouragement of physical activity at home, through online classes and simple exercises with body weight, will prevent the harmful effects on health since increased physical inactivity can lead to an increase in weight and an increased risk of T2DM together with other pathophysiology.

## Figures and Tables

**Table 1:** Sociodemographic characteristics according to serum concentrations of glycated hemoglobin (HbA1c). COVID-Inconfidentes, October to December 2020.

		Hemoglobin Glycated (CI 95%)		
Variables	Total %	Normal (≤6.4%)	High HbA1c (≥6.5%)	*p*-Value
Sex				
Female	52.4 (45.2–59.4)	90.0 (86.6–92.6)	10.0 (7.4–13.3)	
Male	47.6 (40.5–54.8)	95.9 (92.5–97.8)	4.1 (2.2–7.5)	**0.01**
Age Group				
18–34 years	35.1 (30.8–39.7)	99.0 (96.7–99.7)	1.0 (0.29–3.0)	
35–59 years	45.8 (41.2–50.5)	92.2 (89.3–94.3)	7.8 (5.6–10.7)	**<0.001**
≥60 years	19.1 (15.7–23.0)	77.8 (67.8–85.3)	22.2 (14.8–32.3)	
Marital Status				
Married	52.9 (46.8–58.9)	91.2 (88.0–94.7)	8.0 (5.3–12.0)	0.35
Not married	47.1 (41.1–53.2)	94.0 (90.7–96.2)	6.0 (3.8–9.2)	
Race/ethnicity				
White	26.0 (21.2–31.6)	95.8 (93.3–97.4)	4.2 (2.5–6.6)	
Brown	48.1 (41.7–54.5)	94.5 (91.2–96.6)	5.5 (3.4–8.8)	**<0.001**
Black	20.2 (15.6–25.7)	85.8 (77.9–91.3)	14.1 (8.7–22.1)	
Others	5.7 (4.1–7.8)	90.2 (80.1–95.2)	9.8 (4.8–19.0)	
Family income				
≤2 MW	41.2 (35.6–47.1)	92.3 (88.1–95.2)	7.7 (4.9–10.2)	
>2 a ≤ 4 MW	31.4 (26.3–36.9)	93.6 (89.7–96.1)	6.4 (3.9–10.2)	0.81
>4 MW	27.4 (22.3–33.1)	93.2 (88.1–96.8)	6.2 (3.1–11.7)	
Education				
>9 years	69.1 (64.3–73.6)	96.0 (94.2–97.2)	4.0 (2.7–5.8)	**<0.001**
≤9 years	30.9 (26.4–35.7)	86.2 (79.1–90.7)	13.8 (9.3–20.1)	
Overweight				
No	43.5 (37.0–50.2)	96.8 (95.1–97.9)	3.2 (2.1–4.9)	**0.004**
Yes	56.5 (49.8–63.0)	91.6 (87.0–94.6)	8.4 (5.3–13.0)	
Referred Morbidity				
Absence of morbidity	44.5 (37.6–51.6)	99.4 (98.3–99.8)	0.6 (0.9–2.13)	**<0.001**
Presence of morbidity	55.5 (48.3–62.4)	87.3 (83.4–90.3)	12.7 (9.6–16.5)	

PI: physical inactivity; CI: confidence intervals (95%); MW: Minimum wage. “Family income: minimum wage value (2020): BRL 1045.00 ≈ USD 194.25 (1 USD = 5.3797 BRL). Normal glycated hemoglobin (≤6.4%) and high HbA1c (≥6.5%). Overweight (BMI ≥ 25.0 kg/m^2^ if <60 years or BMI ≥ 28.0 kg/m^2^ if ≥60 years). Others in race/ethnicity (yellow, indigenous, or abstained from response) * *p*-value < 0.05 according to Pearson’s chi-square test. The bold indicates the covariates in the table.

**Table 2:** Association of leisure time physical inactivity with high levels of HbA1c. COVID-Inconfidentes, October to December 2020.

	HbA1c (≥6.5%)			
	Univariate OR (IC 95%)	*p*	Multivariate* OR (IC 95%)	*p*
Physically active	1.00		1.00	
Physically inactive	3.36 (1.86–6.08)	**<0.001**	2.62 (1.31–5.24)	**0.007**

Note: Diabetes mellitus (DM). Odds ratios (OR) and 95% confidence intervals (95%CI) from logistic regression. 1. Explanatory variable was individuals who were physically inactive at leisure (<150 min of moderate PA or <75 min of vigorous activity). 2. Outcome variable: normal serum glycated hemoglobin (HbA1c) levels (≤6.4%) and high levels of HbA1c (≥6.5%). * Directed acyclic graph (DAG) was used to support the theoretical model for multivariate analysis. Multivariate regression adjusted according to directed acyclic graph according to minimum and sufficient variables: sex, age, family income, race/ethnicity, and referred morbidity. *p* < 0.05 were considered statistically significant. n = 1685 participants. The bold indicates the covariates in the table.

## Data Availability

The datasets generated and/or analyzed as part of the current study are not publicly available due to confidentiality agreements with subjects. However, they can be made available solely for the purpose of review and not for the purpose of publication from the corresponding author upon reasonable request.
